# Career decision making in undergraduate medical education

**DOI:** 10.36834/cmej.69220

**Published:** 2020-07-15

**Authors:** Shama Sud, Jonathan P. Wong, Laila Premji, Angela Punnett

**Affiliations:** 1 Department of Paediatrics, SickKids Hospital, University of Toronto, Ontario, Canada

## Abstract

**Background:**

It is unclear how medical students prioritize different factors when selecting a specialty. With rising under and unemployment rates a novel approach to career counselling is becoming increasingly important. A better understanding of specialty selection could lead to improved career satisfaction amongst graduates while also meeting the health care needs of Canadians.

**Methods:**

Medical students from the University of Toronto participated in a two-phase study looking at factors impacting specialty selection. Phase I consisted of focus groups, conducted independently for each year, and Phase II was a 21-question electronic survey sent to all students.

**Results:**

Twenty-one students participated in the focus group phase and 95 in the survey phase. Primary themes related to career selection identified in Phase I in order of frequency included personal life factors (36), professional life factors (36), passion/interest (20), changing interests (19) and hidden curriculum (15). The survey phase had similar results with passion (83), lifestyle (79), flexibility (75), employment opportunities (60) and family (50) being ranked as the factors most important in specialty selection.

**Conclusion:**

Personal factors, professional factors and passion/interest may be key themes for medical students when deciding which specialty to pursue. Targeting career counselling around these areas may be important.

## Abstract (fr)

**Contexte:**

On ne comprend pas bien comment les étudiants en médecine priorisent différents facteurs au moment de choisir une spécialité. Étant donné la hausse des taux de sous-emploi et de chômage, il devient de plus en plus important d’adopter une nouvelle approche d’orientation professionnelle. Une meilleure compréhension choix d’une spécialité pourrait accroître le niveau de satisfaction des diplômés en ce qui a trait à leur choix de carrière tout en satisfaisant aussi les besoins en soins de santé des Canadiens et Canadiennes.

**Méthodes:**

Les étudiants en médecine de l’Université de Toronto ont participé à une recherche comportant deux phases examinant les facteurs influençant le choix d’une spécialité. La Phase I comportait des groupes de discussion dirigés indépendamment pour chaque cohorte tandis que la Phase II consistait en un sondage électronique de 21 questions transmis à tous les étudiants.

**Résultats:**

Vingt et un étudiants ont participé à la phase des groupes de discussion et 95 à la phase du sondage. Les principaux thèmes liés au choix de carrière qui ont été identifiés durant la Phase I sont les suivants (en ordre de fréquence) : les facteurs de vie personnelle (36), les facteurs de vie professionnelle (36), la passion/l’intérêt (20), les champs d’intérêt changeants (19) et le curriculum caché (15). La phase de sondage a donné des résultats semblables. La passion (83), le style de vie (79), la souplesse (75), les possibilités d’emploi (60) et la famille (50) se sont révélés comme étant les facteurs les plus importants dans le choix d’une spécialité.

Conclusion :Les facteurs personnels, les facteurs professionnels et la passion/l’intérêt pourraient être les principaux thèmes que partagent les étudiants en médecine au moment de décider à quelle spécialité ils aspirent. Il semble important de cibler les démarches d’orientation professionnelle sur ces thèmes.

## Introduction

Medical school training programs have a responsibility to ensure that the distribution of graduates meet the health needs of the Canadian population.^1^ Nationally the Royal College of Physicians and Surgeons of Canada reports under- and un-employment for up to 13% of specialists and 14% of subspecialists and recommends a systematic approach to career counseling.^2^ Likewise, the Association of Faculties of Medicine of Canada has articulated the need for adequate support in career advising at the undergraduate level, as the number of unmatched medical students rose from 25 in 2009 to 62 in 2019.^3^^,^^4^

It is unclear how medical students prioritize different factors when selecting a speciality. Previous reports make an association with debt, earning potential, and specialty selection.^5^^–^^7^ Students with greater amounts of debt upon graduating medical school tend to select specialties with greater earning potential. Other studies suggest having greater control over personal lifestyle is important.^8^^–^^10^ There is no clear consensus on whether student membership in subspecialty groups, the assignment of a family physician or subspecialist mentor, or participation in longitudinal clerkships impact career decision making and specialty selection.^11^^-^^14^ Burnout is also an important factor for some medical students.^15^^,^^16^

A better understanding of how medical students decide upon a specialty can facilitate the management of expectations at the undergraduate level, promote the social accountability mandate of training, and foster career satisfaction among trainees. The objective of our study was to identify which factors were most important in guiding specialty selection among medical students from the University of Toronto, and to rank the relative importance of each factor. We found no other study examining how Canadian medical students prioritize multiple factors in the specialty selection process.

## Methods

The Research Ethics Board (REB # 00032825) at the University of Toronto approved this study prior to initiation. We invited medical students from all four years at the University of Toronto to participate in this study. All participants in Phase I provided written consent, and participants in Phase II provided implied consent in agreeing to complete the survey.

We conducted the study in two phases. Phase I took place in the fall of 2016 using a focus group format (Appendix A). We conducted separate focus groups for students from each year of training and investigators in the study were responsible for moderation. We collected demographic information from participants. We recorded the audio component of the focus groups and transcribed them verbatim. Data was analyzed in an iterative manner, using constant comparative analysis based on the grounded theory approach. Three investigators (SS, JW, AP) independently coded and analyzed the data and generated themes and patterns. We discussed discrepancies in themes until we achieved a group consensus. The fourth investigator (LP) performed investigator triangulation to increase the validity of the results.

Phase II of the study was a survey using data from the focus groups to develop a questionnaire with REDCap Research Electronic Data Capture Software®. The survey consisted of 21 different questions asking about medical student specialty selection (Appendix B). We sent the survey via e-mail to all medical students at the University of Toronto in the fall of 2017. We offered a $100 gift card prize to encourage participation and awarded to one of the participants. The investigators populated the data from the online survey into Microsoft Excel, and analyzed it. Descriptive statistics are described as mean ± SD. Proportions are expressed as percentages. The survey served as a form of methodological triangulation generating a second dataset with which to compare findings from the focus group phase of the study.

## Results

### Phase I. Focus group data

Twenty-one students participated in total (Table 1) including nine students in year one, nine students in year two, and three students in year four. Multiple attempts were made to conduct a focus group with students from year three, including offering evening sessions. Unfortunately, the response rate was poor and despite confirmation, students did not attend the meetings. The majority of participants were aged 20-23 (62%) and slightly more than half were female (57%). Two-thirds of students completed an undergraduate degree as their highest level of education prior to beginning medical school (67%). Most of the participants were unmarried (90%) and wanted to stay in Toronto for their postgraduate training (81%).

**Table 1:** Demographic information for participants from Phase I.

Demographic Information	
Age n (%)	
20-23	13 (62)
24-27	7 (33)
32-35	1 (5)
Year of Training n (%)	
Year 1	9 (43)
Year 2	9 (43)
Year 3	0 (0)
Year 4	3 (14)
Gender n (%)	
Male	9 (43)
Female	12 (57)
Education Prior to Medical School n (%)	
Undergraduate Degree	14 (67)
Master’s degree	6 (29)
Professional Degree	1 (5)
Relationship Status n (%)	
Single	19 (90)
Married	2 (10)
Postgraduate Location Preference n (%)	
Toronto	17 (0.81)
Other	2 (0.10)
Unsure	2 (0.10)

**Figure 1:**
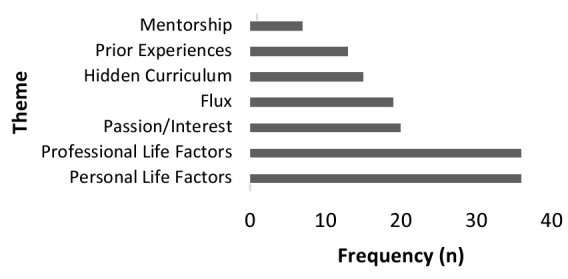
Frequency of theme occurrence

Seven primary themes emerged: professional life factors; personal life factors; passion/interest; changing interests; hidden curriculum; prior experiences; and mentorship (See Figure 1). Within the seven primary themes, an additional 14 subthemes were identified. See Table 2.

**Table 2:** Summary of primary themes and sub-themes

Theme/Sub-theme	Frequency	Example
*Professional Life Factors (36)*		
Flexibility	19	“Flexibility—with that is your opportunity to do things other than just your clinical practice. “
Job Market	8	“One for me is really job opportunities at the end of it all.”
Income	6	“For me, it was about medical school and about the time that I wouldn’t be making money in terms of 4 years of medical school plus residency...”
Research Opportunities	3	“If I see a lot of interesting research in a specialty…I can see whatever part of this research is coming to fruition and be a part of that.”
*Personal Life Factors (36)*		
Lifestyle	12	“…lifestyle and what you want outside of medicine is a big factor.”
Family Commitments	9	“I am starting in my thirties, and if I go into a longer residency program, then I won’t really be earning an income until I am in my forties, and I have a family.”
Location	10	“For me it’s (location) only. When I look at lifestyle, I don’t see myself living outside (location).”
Maternity Leave/Pregnancy	5	“I think it’s different for women. For us, when can we get pregnant and start a family? Women are more affected by the length of the program.”
Passion/Interest	20	“In general, making sure you are going to like what you want to do forever. “
Changing Interests	19	“Even for me, I haven’t had exposure to all of the specialties yet, but I find my interests have changed dramatically from the when I began medical school. So, I imagine that once I get exposure to many more specialties, my interests will change a lot.”
*Hidden Curriculum (15)*		
Prestige	4	I think prestige plays into a factor in what type of career you want. I did my Master’s in chemistry and all the people that are hired on as faculty for a science or academic position have to have come from Stanford or Harvard or all these big things. So if I did a fellowship at Harvard, I could get hired for a faculty position at U of T.
Stigma	11	“Some people say “just family medicine”, including those who want to go into it. So there definitely seems to be an ingrained stigma about it.”
*Prior Experiences (13)*		
Prior Academic	9	“Prior to coming into medicine, I was pretty set on what I wanted to do… because I did my Master’s in Public Health.”
Prior Personal	4	“At one point I was thinking about cardiology because I grew up with a heart condition.”
Mentorship	7	“I think so far one of the big motivators towards the specialties I’ve been interested in has been the mentors who have been in my life.”

The four subthemes within professional life factors (36) included *flexibility* (19), *job market* (8)*, income* (6) and *research opportunities* (3):

- “I have this fear of someone in my family being sick and my not being able to take care of them because of work. You never know what life is going to throw at you. And that’s one of the reasons why I value flexibility.”
- “…Three years out they still don’t have a job and so they enter a Ph.D. program, not because they want to, but because they have to fill in the time.”
- “In terms of debt, I would like to know, going into residency, if I am likely to get a job afterwards. I said I was interested in surgery before, but I’ve heard it is not likely that I would get a surgery position in Ontario or in Canada. Consequently, my chances of getting rid of that debt would be harder.”
- “I’m interested in researching surgical error and mitigating surgical error…”

The four subthemes within personal life factors (36) included *lifestyle* (12), *family commitments* (9), *location* (10) and *maternity leave/pregnancy* (5):

- “Lifestyle would be a big one. I think that is one of the factors which divides people as to whether they want to pursue Family Medicine or something like Surgery which is much more time-consuming...”
- “I’m interested in Emergency Medicine right now and I think about how the shift work would affect the quality of time with the family.”
- “… I have to live in (location), I can’t live anywhere else. I lived in (location) for my first year of undergrad and I almost went crazy…”
- “…for women, we know that we are the ones getting pregnant, we are probably thinking more about what would come after that (residency).”

Passion/interest (20 items), although a key theme, was vocalized less frequently than professional and personal life factors as playing a significant role in the decision-making process:

- “At the end of the day, I think I made more of an emotional decision. I decided on something I really liked….”

Changing interests was found to be a primary theme with a difference between changing interests that were expected versus changing interests leading to anxiety and confusion (19):

- “Even for me, I haven’t had exposure to all of the specialties yet, but I find my interests have changed dramatically from the when I began medical school. So, I imagine that once I get exposure to many more specialties, my interests will change a lot.”
- “I find it a little overwhelming. When you go in you are bombarded with so much information.”

The hidden curriculum (15 items) and the subthemes of *prestige* (4) and *stigma* (11) emerged with concerns being voiced around family medicine in particular:

- “So, when someone asks you what are you thinking of going into, neurosurgeon sounds really cool, but family doctor doesn’t sound that cool.”
- “With regard to family medicine, there is the feeling that you should be striving for more. There is the sense that family medicine is the default in case you don’t get your specialty.”

Two sub-themes arose under the primary theme of prior experiences (13 items), including *prior academic* (9) and *prior personal* (4):

- “I had a few ideas when I came in. My undergrad was in neuroscience, so part of me thought about neurology.”
- “I came in with an interest in endocrinology from personal experience. I just thought of it very fast.”

Mentorship (7 items) was important for some students:

- “More than anything for me, it’s the residents. The residents are so close to being in our shoes. The way they came to their decisions helps me to evaluate my choice.”

When asked which additional resources helped in the decision-making process, many students cited wanting a list of mentors from different specialties and additional information on the various specialties:

- “I would like to see a list of mentors and their specialties for all four years as well as some information on their specialties.”

### Phase II. Survey data

Ninety-five students participated in the second phase of the study (See Table 3). Although students from all years were invited to participate, the vast majority were from year four (*n =* 93). There were two students from year three that participated. Approximately half of the students were female (53%). One-third of the students were single (29.5%), 21.1% were engaged or married, and an additional 50% were in a relationship. Most students had completed an undergraduate degree prior to starting medical school (72%) while about a quarter (24.2%) had completed a master’s degree or Ph.D. A large proportion of students came from families with a household income of greater than $100 000 (41%). Of the 95 participants, 10 had at least one immediate family member (parent and or sibling) who was a physician (10.5%). The Ontario School Assistance Program was the most commonly cited source of funding for medical school (84%), followed by a line of credit (77%), parental support (57%) and personal savings (31%).

**Table 3:** Demographic information for participants in Phase II.

Demographic Information *n* = 95	
Age (mean ± SD, range)	26 ± 2.3 (23-35)
Year 4 (n (%))	93 (98)
% Female/Male	53/47
Relationship Status (%)	
In a relationship	50
Engaged	9.5
Married	11.6
Single	29.5
Partner in the Same City (%)	72
Have Children (%)	4
Training Before Medical School (%)	
Undergraduate	72
Masters	20
Ph.D.	4.2
Other	3
High School Household Income (%)	
<25 000	9.5
25-50 000	10.5
50-100 000	39
100-150 000	21
>150 000	20

**Figure 2:**
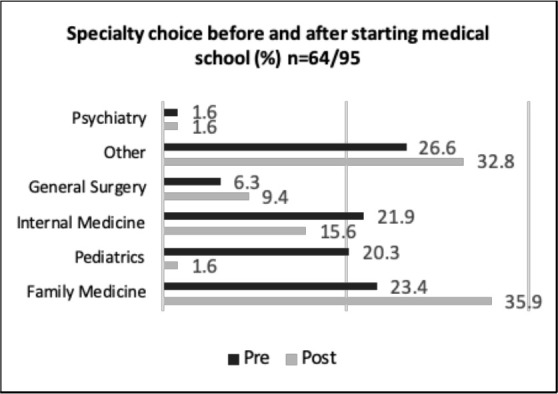
Change in specialty choice before and after beginning medical school

Prior to beginning medical school, two-thirds of students had an idea of which specialty they wanted to pursue and the same proportion of students changed their mind. The biggest changes were with students interested in pursuing Paediatrics (decreased from 20.3% to 1.6%) and family medicine (increased from 23.4% to 35.9%) See Figure 2. Reasons cited for changing specialties included experiences in medical school (51%), medical school curriculum (39%), mentorship (34%), shadowing (33%), peer attitude toward specialty (13%), and other (16%). When asked about the experience of career selection, 58% of students found it to be as expected with many changes during medical school, 56% found it to be associated with a lot of stress and anxiety, and 54% found it to be exciting; some students selected more than one response.

**Figure 3:**
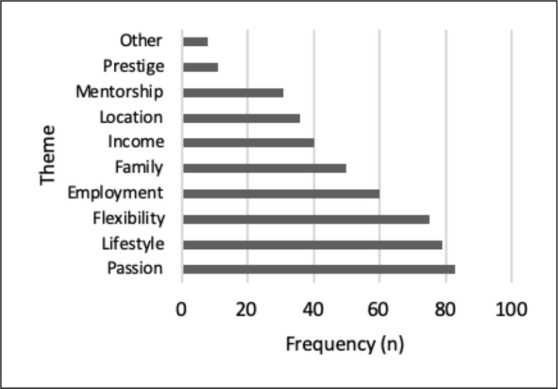
Top 5 factors influencing specialty choice

We asked students to rank which five factors were most important (from 1 to 5 with 1 being the most important and 5 being the least) in guiding specialty selection (Figure 3). Passion was the most frequently documented factor (*n =* 83), followed by lifestyle (*n =* 79), flexibility (*n =* 75), employment opportunities (*n =* 60), family (*n =* 50), income (*n =* 40), location (*n =* 36), mentorship (*n =* 31), and prestige (*n =* 11). Eighty-one students (85%) agreed that specialties were viewed differently, with 61 (65%) stating that being a specialist is viewed as more prestigious. Some students felt all specialties were viewed as more valuable than family medicine, “There is a general tendency within medicine to look down on general practitioners...There's a lot of value put on surgical and lifestyle specialties.” Personal resources such as mentors (73%), shadowing (65%), longitudinal experiences (54%) and peer mentoring (51%) were used most frequently to guide specialty selection, with websites (23%) and student counselling services (16%) being used less commonly.

## Discussion

In this study examining which factors are important when deciding on a specialty to pursue, we found that medical students considered personal and professional life factors (lifestyle and flexibility) and passion/interest to be the most important. This was reflected in both Phase I and Phase II of the study, although passion for the field was identified as the most important factor amongst the survey respondents, the vast majority of whom were fourth year students. This may be in part because they have had more time to identify where their interests lie after completing a significant amount of their clinical training and electives. This is in keeping with a previous study done by Clinite and colleagues who found that students ranked “enjoying the type of work I am doing” as the most important lifestyle domain and “being satisfied with the job” as the most important specialty characterisitic.^17^ Other studies have also found a trend for students to select specialties with greater control over lifestyle.^8^^,^^9^

The perceptions of the local education culture were notable. Many students felt that family medicine was undervalued compared to other specialties, despite efforts undertaken at the University of Toronto to promote interest in family medicine (such as a longitudinal family medicine clinical experience in pre-clerkship). This finding has been previously described in the literature with students reporting that some specialty preceptors disparage family medicine.^18^ A study done in Switzerland found that a more cohesively developed family medicine curriculum taught by a small group of staff may lead to a more positive view of family medicine.^19^ The broad exposure to specialists through both the classroom and clinical training at tertiary/quaternary care centers in Toronto may impact students’ perceptions. While prestige was not a prominent theme and many students selected family medicine as a career choice, it is difficult to know if and how the hidden curriculum and education culture impacted the decision-making process.

Many students reported feelings of anxiety and stress around the career decision-making process suggesting there is increased need for support around this area. We shared the results of our study with the Associate Dean of Student Affairs at the University of Toronto and multiple initiatives have since been implemented. For example, the MD program has initiated a “Check your Pulse” student check-in with personal counsellors and has recruited clinical faculty members to play a larger mentorship role in the career decision making process for students.

Personal resources including staff and peer mentorship, shadowing and longitudinal experiences appear to be the most helpful for students in their decision-making process. In support of this, pre-clerkship students at the University of Toronto are now provided one day per week free of structured curriculum time to engage in shadowing and mentoring experiences to help with career development. Third year clerks also have the opportunity to complete a two-week elective aimed at promoting earlier career exploration.

Contrary to previous studies, we did not find that students selected specialties based on their level of debt.^5^^–^^7^ It may be that Canadian students have less debt or are less concerned about their debt than students in the United States. It may also suggest that students are prioritizing passion for a specialty over income.

## Limitations

There are multiple limitations to our study. Primarily, this was a single center study and the results may not be generalizable to other training programs due to cultural and structural differences between centers. In addition, despite many attempts to conduct focus groups with the third-year students we were unable to capture their insights. This is likely a result of limited flexibility and time in their clerkship schedule. The sample size was relatively small for the focus groups making it tenuous whether these results could be generalizable to the entire student body. It is reassuring, however, to see that the results from the first phase of the study were mirrored quite closely in the second phase of the study. Phase II of the study included mostly fourth year students, perhaps in part reflecting their active thinking about specialty choices as they complete electives and prepare for the residency match in addition to their more flexible schedules. As with any focus group or survey study, there may be a selection bias for those choosing to participate.

### Future directions

We hope to follow a longitudinal cohort over the four years of medical school to further describe the changing feelings and perceptions related to specialty selection. We are also interested in evaluating a multi-center sample to see how differences in culture and location may affect students’ career decision-making.

## Conclusions

Personal and professional life factors (in particular, lifestyle and flexibility) as well as passion for and interest in the work of a specialty appear to be highly important considerations for medical students at the University of Toronto when deciding which specialty to pursue. Targeting resources accordingly may be important to support student decision-making and optimal career satisfaction among trainees. Medical schools can bolster their efforts to improve faculty mentorship by collaborating with their alumni associations, creating faculty positions for clinicians focused on career mentorship and through appropriate faculty development. In addition, attention to the education culture and ensuring that family medicine or other generalist fields are not undervalued is critical. Given the shortage of family physicians in Canada and the societal responsibility that medical schools have in training generalists there may be a need to positively highlight or showcase this specialty to attract and retain trainees in this area.^20^ Medical schools may also consider developing a formal and longitudinal career exploration and preparedness curriculum based on career development theory to identify and build on individual students’ values, strengths and interests.^21^ Further research including evaluation of such curricula will be pivotal to developing the most effective educational strategies.

## Appendix A Questions used in focus groups

1. What specialty did you think you wanted to pursue before coming to medical school (for years 2-4)?
2. What are some of the factors that come to mind when thinking about which medical specialty you want to pursue?
  - What about personal factors (relationship, age, children, number of years of education before medical school, debt, family expectations, location, support system)?
  - What about specialty specific factors (length of training, culture, duration of training, interest in the specialty, prestige, amount of call, work-life balance, anticipated stress level, job opportunities, culture, anticipated salary, opportunity to do research)?
  - What about funding cuts?
  - Why?
3. Which factor is the single most important? Second? Third?
4. How have these factors changed since you began medical school?
5. How do your peer’s or your partner’s decisions impact your decision-making process?
6. How have the mentors that you’ve had influenced your decision? Early clinical experiences?
7. What resources do you know of or have you used to help make your decision?
8. What type of resource/experience do you think is not available that would help you make your decision more easily?
9. What specialty did you think you wanted to pursue before coming to medical school (for years 2-4)?
10. What are some of the factors that come to mind when thinking about which medical specialty you want to pursue?
  - What about personal factors (relationship, age, children, number of years of education before medical school, debt, family expectations, location, support system)?
  - What about specialty specific factors (length of training, culture, duration of training, interest in the specialty, prestige, amount of call, work-life balance, anticipated stress level, job opportunities, culture, anticipated salary, opportunity to do research)?
  - What about funding cuts?
  - Why?
11. Which factor is the single most important? Second? Third?
12. How have these factors changed since you began medical school?
13. How do your peer’s or your partner’s decisions impact your decision making process?
14. How have the mentors that you’ve had influenced your decision? Early clinical experiences?
15. What resources do you know of or have you used to help make your decision?
16. What type of resource/experience do you think is not available that would help you make your decision more easily?

## Appendix B. Survey questions

**Demographics:**

1. What year of medical school are you in?

(a) 1 (b) 2 (c) 3 (d) 4+

2. What is your age in years?

3. What is your gender?

(a) Male (b) Female (c) Other ________

4. What is your relationship status?

(a) Single (b) In a relationship (c) Engaged (d) Married

If the answer to question 4 was (b),(c), or (d) please answer question 5; otherwise move on to question 6

5. Do you and your partner live in the same city?

(a) Yes (b) No (c) Not Applicable

6. Do you have children?

(a) Yes; if yes how many? ______________ (b) No

If the answer to question 6 was ‘Yes’ please answer question 7; otherwise move on to question 8

7. How many children do you have?

(a) 1, (b) 2, (c) 3, (d) 4 +

8. What was your level of education prior to beginning medical school?

(a) Undergraduate degree (b) Master’s degree (c) Ph.D. (d) Other professional degree (ie. nursing, pharmacy, social work)

9. What was the approximate household income in your high school years?

(a) ≤25000 (b) 25000-50000 (c) 50000-100000 (d) 100000-150000 (e) ≥150000

10. Are any of your immediate family members physicians?

(a) yes; if so who?___________________ (b) no

11. What types of financial support do you have for completing medical school? Choose all that apply.

(a) OSAP (b) Line of credit (c)Personal funds (d) Parental support

**Main survey:**

12. Did you have an idea of which residency specialty you would pursue before starting medical school?

(a) Yes (b) No

If the answer to question 12 was “yes” please answer questions 13 and 14; otherwise move on to question 15

13. What was the specialty you wanted to pursue?

(a) Anesthesiology (b) Family medicine (c) General surgery (d) Internal medicine (e) Pediatrics (f) Psychiatry

(g) Other ______

14. Has your choice of specialty changed since starting medical school?

(a) Yes (b) No

If the answer to question 14 was “yes” please answer questions 15 and 16; otherwise move on to question 15

15. What specialty are you currently interested in pursuing?

(a) Anesthesiology (b) Family medicine (c) General surgery (d) Internal medicine (e) Pediatrics (f) Psychiatry

(g) Other ______

16. Why has your choice in specialty changed? (Select as many as appropriate)

(a) Exposure to medical school curriculum

(b) Medical school

(c) Mentorship

(d) Peer attitude towards specialty

(e) Shadowing

(f) Other: __________

17. The process of career selection has been (choose all that apply)

(a) An exciting opportunity

(b) As expected with many changes during medical school

(c) Associated with a lot of stress and anxiety

(d) Other

18. Please rank the top 5 factors (1 being most important and 5 being least important) influencing your specialty choice at this time from the list provided below:

__Employment opportunity/job market

__Family and/or partner commitments

__Income

__Job flexibility/ autonomy

__Lifestyle

__ Location

__Mentorship

__Passion/fit

__Prestige

__Other: __________

19. Do you think different specialties are valued differently?

(a) yes (b) no

If your answer to question 19 was “yes” please answer question 20; otherwise move on to question 21

20. From your perspective, how are specialties in medicine valued differently?

21. Are there any resources you have used to help your residency specialty decision? (Select as many as appropriate)

(a) Faculty mentors

(b) Longitudinal experiences

(c) Peer mentors

(d) Shadowing/Early Clinical Experiences

(e) University provided services (ie OHPSA)

(f) Websites (ie AFMC)
